# La^3+^’s Effect on the Surface (101) of Anatase for Methylene Blue Dye Removal, a DFT Study

**DOI:** 10.3390/molecules27196370

**Published:** 2022-09-27

**Authors:** Ximena Jaramillo-Fierro, Sneyder Gaona, Eduardo Valarezo

**Affiliations:** Departamento de Química, Facultad de Ciencias Exactas y Naturales, Universidad Técnica Particular de Loja, San Cayetano Alto, Loja 1101608, Ecuador

**Keywords:** DFT calculations, anatase, lanthanum, adsorption, methylene blue

## Abstract

Density functional theory (DFT) is a widely used method for studying matter at the quantum level. In this study, the surface (101) of TiO_2_ (anatase phase) was considered to develop DFT calculations and explain the effect of lanthanum ion (La^3+^) on the electronic properties, adsorption capacity, and photocatalytic activity of this semiconductor. Due to the presence of the La^3+^ ion, the bandgap energy value of La/TiO_2_ (2.98 eV) was lower than that obtained for TiO_2_ (3.21 eV). TDOS analysis demonstrated the presence of hybrid levels in La/TiO_2_ composed mainly of O2*p* and La5*d* orbitals. The chemical nature of the La-O bond was estimated from PDOS analysis, Bader charge analysis, and ELF function, resulting in a polar covalent type, due to the combination of covalent and ionic bonds. In general, the adsorption of the methylene blue (MB) molecule on the surface (101) of La/TiO_2_ was energetically more favorable than on the surface (101) of TiO_2_. The thermodynamic stability of doping TiO_2_ with lanthanum was deduced from the negative heat-segmentation values obtained. The evidence from this theoretical study supports the experimental results reported in the literature and suggests that the semiconductor La/TiO_2_ is a potential catalyst for applications that require sunlight.

## 1. Introduction

The density functional theory (DFT) is a theory developed for the calculation of many-body systems, especially formed by large numbers of atoms and molecules. It is an important theory in the field of molecular physics since it allows calculating electronic, atomic, and molecular interactions [1]. The origins of this theory lie in a model developed by Llewellyn Thomas and Enrico Fermi in the late 1920s. However, it was not until the mid-1960s that contributions from Pierre Hohenberg, Walter Kohn, and Lu Sham established the theoretical formalism on which the currently used method is based [2].

The traditional methods within the theories of the electronic structure of matter, in particular the Hartree-Fock theory and derivatives of this formalism [3], are based on a multi-electron wave function. Although this resolution of the Schrödinger equation makes it possible to accurately describe the behavior of very small systems, its predictive ability is limited by the fact that the Schrödinger equations are too complex to solve numerically, much less analytically [1,4]. The density functional theory (DFT) reformulates the problem in order to obtain, for example, the distribution of energy and electrons of the ground state, working with the electron density functional instead of the wave function. One advantage is that the density is a much simpler quantity than the wave function and therefore easier to calculate. In practice, much more complex systems are accessible: the wave function of a system of N electrons depends on 3N variables, while the electron density only depends on three variables [5].

The DFT applied to electron systems is an alternative variational procedure to the solution of the Schrödinger equation, where the functional of the electronic energy is minimized with respect to the electronic density. It is one of the most widely used methods in quantum calculations of the electronic structure of matter, in both condensed matter physics and quantum chemistry [6]. The main advantage of DFT is that its equations are much simpler to solve than the many-body equations of quantum mechanics or other approximations, which makes it possible to work with larger systems and calculate more of their properties. With this theory, it is possible to perform simulations with a few thousand atoms. Recently, the use of the DFT method has been considered to study the molecular interaction of various organic compounds, including dyes, with matrices of different chemical natures, such as hydrogels, porous nanoadsorbents, nanomembranes, and pure or doped metal oxides [7,8,9,10]. One of the main objectives of these studies has been to explain at the molecular level the mechanisms of surface adsorption of such compounds to promote future technological and environmental applications [11,12,13,14].

Synthetic dyes are used worldwide in a wide range of products in the food, cosmetic, pharmaceutical, paper, and textile industries [15]. These dyes are stable and non-biodegradable, are designed to maintain color on various materials, and are resistant to water, soap, and oxidizing agents [16]. Wastewater containing dyes, when discharged in water effluents, inhibits the penetration of sunlight and reduces photosynthetic action [17]. Waters contaminated with dyes are characterized by having a high chemical oxygen demand (COD), high pH, and biological toxicity [18]. Most synthetic dyes are toxic and carcinogenic, so if wastewater containing these substances is not treated effectively, it can be responsible for serious environmental damage and also pose a threat to public health [19]. Synthetic dyes can be classified as anionic, cationic, and non-ionic. Methylene blue (MB), classified as a cationic dye, is one of the dyes widely used in the industry to dye cotton, wood, and silk [20]. Methylene blue is toxic: if ingested in large quantities, it produces harmful effects such as gastritis, severe headache, painful urination, methemoglobinemia-like syndromes, profuse sweating, mental confusion, and respiratory toxicity [21,22].

In general, wastewater contaminated with synthetic dyes is treated by physicochemical processes such as ultrafiltration, coagulation, and photooxidation, but these treatments have limitations such as the formation of toxic by-products, for their operation requires large amounts of energy and is not adapted to the wide range of dyes that exist in the market [23]. The disadvantages of these methods have motivated us to investigate alternative methods for the removal of dyes, one of which is adsorption, a technique that has been widely used for the removal of dyes where a solid adsorbent is used to attract the dye molecule and finally lead to its elimination from the aqueous medium. The material most used as an adsorbent is activated carbon, although the use of metal oxides has also been explored [24].

Titanium dioxide (TiO_2_), also called titanium oxide (IV), is a compound that is created naturally when titanium reacts with oxygen in the air. In its oxide form, titanium is found in minerals in the earth’s crust. Titanium dioxide is an easily synthesized material widely used in energy-related applications, such as the photocatalytic splitting of water and the conversion of solar energy into electricity. This semiconductor metal oxide is used to construct electron transport channels due to their appropriate bandgaps (3.2 eV) and efficient electron mobilities. In addition, this oxide is used for surface anti-corrosion, water purification, and organic contaminant removal due to its tunable surface and structural functionality [25]. In nature, there are three main forms of TiO_2_, brookite (orthorhombic structure), anatase (tetragonal structure) and rutile (tetragonal structure). The stable polymorphs of TiO_2_ are rutile and anatase, while bulk rutile is more stable. Small TiO_2_ nanoparticles with a large surface area are typically composed of the anatase polymorph thanks to its low surface energy [26]. Both rutile and anatase are produced on a large scale industrially and are used primarily as catalysts or surface pigments. Its annual production was 100 thousand tons in the year 2021 [27].

Anatase is one of the three crystalline forms of titanium oxide. Due to the elongated shape of its crystals, it has also been called octahedrite, since the elongated tetragonal bipyramids form an octahedron. This oxide occurs in small crystals that can only be seen through a magnifying glass or binocular microscope. However, the variability of its crystalline habit and the richness in its faces, together with its lively brightness and multiple colors, make it an interesting mineral. TiO_2_ anatase nanoparticles in their equilibrium form have a truncated bipyramid shape with eight surfaces (101) and two surfaces (001). The most stable and most commonly occurring anatase crystallographic surface is (101) [21]. In general, ordinary anatase is composed of crystal surfaces (101) with higher surface energy, while crystal surfaces (001) with higher reactivity are almost non-existent due to their low surface energy [28].

Rare earths are the common name for 17 chemical elements: scandium, yttrium, and the 15 elements of the lanthanide group, among which include cerium (Ce), europium (Eu), gadolinium (Gd), lanthanum (La), neodymium (Nd), praseodymium (Pr), samarium (Sm), and others [29]. Lanthanides are recognized as the activators of the luminescence phenomenon with the greatest potential to obtain an emission in the visible region of the electromagnetic spectrum. For this reason, the development of new TiO_2_-based materials doped with rare earths at the +3 energy level (Ce^+3^, Eu^+3^, Gd^+3^, La^+3^, Nd^+3^, Pr^+3^ and Sm^+3^) is interesting in order to reduce the band gap energy (Eg) of said materials and take advantage of the potential of visible light while inhibiting the recombination of the electron-hole pair (*e*^−^/*h*^+^) [30].

Previous experimental results have shown that the incorporation of La on the lattice of TiO_2_ induces the narrowing of its bandgap and also promotes photocatalytic activity [31,32,33]. Yang et al. [33] have investigated the photocatalytic activity of La-doped TiO_2_ in converting methanol into methyl formate and found that the rare earth La on the surface of TiO_2_ can form impurity levels in the valence band and conduction band, reduce the band gap, change surface electron states, and decrease the potential energy for surface hydroxyl formation, so La/TiO_2_ is more active than TiO_2_ in terms of energy barriers of the transition states. However, the fact that, to date, there are no studies on the synergistic effect of the La^3+^ ion on the surface anatase (TiO_2_) in terms of its adsorption capacity of methylene blue motivated the authors to carry out this study, which aims to use the computational calculations of density functional theory (DFT) to determine the effect that the rare earth lanthanum at its energy level +3 (La^3+^) would have on the surface (101) of titanium dioxide in its anatase form for the removal methylene blue dye. The novelty of this theoretical study lies in the fact that it was possible to demonstrate that the La/TiO_2_ semiconductor can efficiently combine adsorption and photocatalysis processes to remove the MB dye.

## 2. Results

### 2.1. Optimization of La/TiO_2_

**Figure 1:**
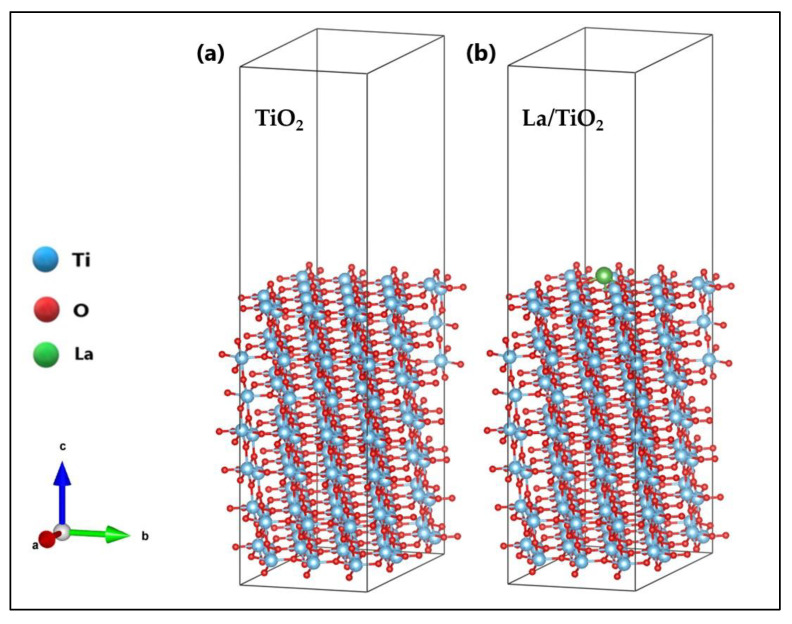
Optimized surface (101) of (**a**) TiO_2_ and (**b**) La/TiO_2_.

The crystal structure of TiO_2_ (anatase phase) with a tetragonal Bravais lattice and I41/amd(141) space group [34,35] was optimized in a previous study [7]. The obtained bulk lattice parameters for the anatase structure were a = 3.821 Å and c = 9.697 Å which are in close agreement with the experimental values (a = ~3.785 Å and c = ~9.514 Å) reported in the literature [36,37,38,39]. In this study, lanthanum was placed on the surface (101) of TiO_2,_ and the effect of this ion on methylene blue (MB) dye removal was investigated. Both the TiO_2_ and La/TiO_2_ surfaces were first subjected to a relaxation process, during which the surface layer of atoms was allowed to relax freely while the other layers immediately below remained fixed. The surface (101) of both TiO_2_ and La/TiO_2_ after relaxation is shown in Figure 1a,b, respectively.

As Figure 1 shows, the La ion formed bonds with three O atoms on the TiO_2_ surface. La-O bond lengths ranged from 2.19 Å to 2.43 Å with an average of 2.31 Å, and La-O-Ti angles ranged from 90.30° to 114.29° with an average of 102.30°; consequently, it is not possible to find elements of symmetry in the La polyhedron. Other authors [40] have also reported this type of geometric distortion. Table 1 shows relevant bond lengths and angles on the surface of La/TiO_2_.

In addition, the surface energy value ($\gamma_{s}$) of the La/TiO_2_ surface with a vacuum distance of 20 Å from the surface (101) was determined to be 0.054 eV/Å^2^ (1.10 J/m^2^). This value calculated using Equation (1) is similar to the previously mentioned surface energy value for the surface (101) of TiO_2_, which was 0.058 eV/Å^2^ (1.21 J/m^2^) [7].

The adsorption energy (Δ*E_ads_*) of the La ion on the surface (101) of the anatase oxide was calculated using Equation (2), where $E_{sorb/surf}$ is the energy of the supersystem resulting from the adsorption of the La ion on the surface of TiO_2_ (eV), $E_{surf}$ is the energy of the free oxide (eV), and $E_{sorb}$ is the energy of the isolated La ion in a vacuum (eV). The adsorption energy value of the La ion on the surface (101) of TiO_2_ was calculated to be −858.30 kJ/mol. The adsorption of the La ion involved the formation of new chemical bonds and, therefore, the occurrence of a chemisorption process [41].

### 2.2. Electronic Structure of La/TiO_2_

**Figure 2:**
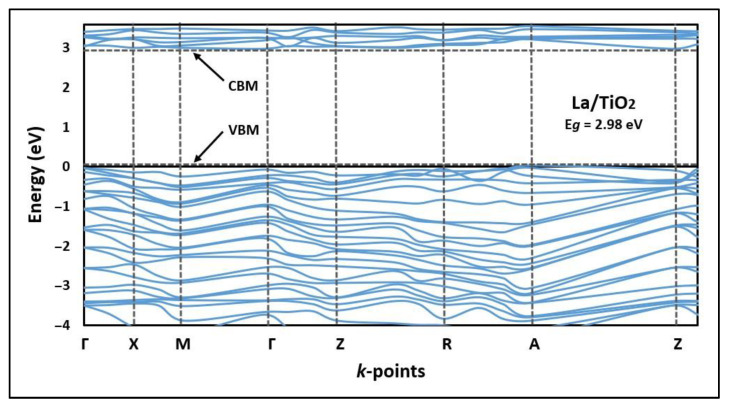
Band structure (BS) of La/TiO_2_ along the high symmetry directions in the Brillouin zone.

The lines and points of high symmetry projected in the first Brillouin zone [42] and the electronic band structure (BS) calculated by the GGA + U method for La/TiO_2_ are shown in Figure 2.

Figure 2 shows that the indirect bandgap energy value of the La/TiO_2_ structure was 2.98 eV, which is 0.23 eV lower than the indirect bandgap energy value we previously reported for the structure of TiO_2_ [7]. Therefore, compared to TiO_2_, La/TiO_2_ would require a lower electron transition energy from the valence band (VB) to the conduction band (CB) [43].

**Figure 3:**
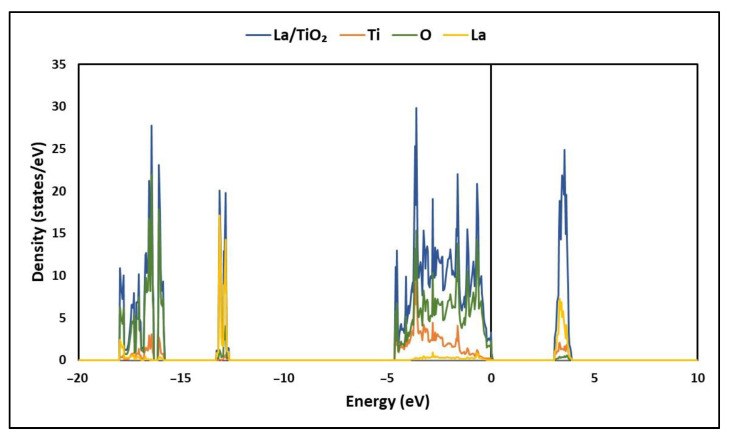
Total density of states (TDOS) of La/TiO_2_.

On the other hand, the density of states (DOSs) was calculated to estimate the chemical nature of the bonds present in the La/TiO_2_ structure [22]. Figure 3 shows the total density of states (TDOS) of the La/TiO_2_ structure. In this figure, it can be seen that the energy levels corresponding to the conduction band (CB) and the valence band (VB) of La/TiO_2_ are slightly denser than those reported for TiO_2_ in our previous study [7], probably due to the contribution of hybrid levels, represented mainly by La5*d* and O2*p* orbitals [33].

The TDOS of La/TiO_2_ shows two main regions: a lower region corresponding to the lower valence band (VB), from about −18 to 0 eV and an upper region corresponding to the upper conduction band (CB) from about 3 to 4 eV. In the Valencia band, it is evident that the main contribution is made by O, while both Ti and La contribute mainly to the conduction band. In the La/TiO_2_ structure, the O atom borders the valence band maximum (VBM), while the Ti atom borders the conduction band maximum (CBM).

**Figure 4:**
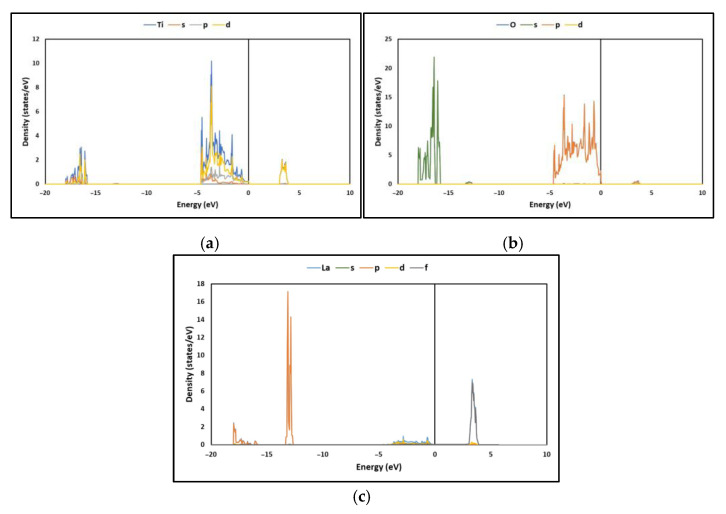
Partial density of states (PDOS) of (**a**) Ti, (**b**) O and (**c**) La.

In addition, the partial density of states (PDOSs) of La/TiO_2_ is displayed in Figure 4a–c. These figures show that in the energy range of −18 to −16 eV, the TDOS results mainly from the O2*s* orbital, with some contribution from the Ti3*p*, Ti3*d* and Ti4*s* orbitals. In the energy range from −5 to −0 eV, TDOS results mainly from the Ti3*d* and O2*p* orbitals. In the 3 to 4 eV energy region, which is above the Fermi level, the total DOS results mainly from the Ti3*d* and O2*p* orbitals. These results agree with those reported in the literature [34]. The PDOS of the O and La atoms on the surface (101) of the La/TiO_2_ structure are displayed in Figure 4b,c, respectively, where two types of hybridizations are evident. The first type of hybridization arises in the energy range from −5 to 0.1 eV due to the overlap of the La5*d* and O2*p* states. The relative amplitude of the peaks shown in this region indicates that these orbitals are delocalized, suggesting ionic bonding. The second type of hybridization arises in the energy range of −18 to −16 eV and around −13 eV due to the overlap of the La5*p* and O2*s* states. The relative narrowness of the peaks shown in these regions indicates that these orbitals are localized, suggesting covalent bonding. Consequently, the results suggest that the La-O bond could be of the polar covalent type, resulting from the combination of ionic and covalent bonds. These results agree with those informed in the literature [44].

To confirm the ionicity of the La-O bond described in the DOS analysis, a Bader charge analysis was also performed on the La/TiO_2_ structure [45]. This analysis is useful since it allows for determining the ionicity of a chemical bond from the charge transfer between the atoms that participate in said bond [46,47,48]. In the La/TiO_2_ structure, the net charge of Ti (+2.5*e*) was 1.5*e* less than its formal +4*e* charge, while the O atom had a negative charge of −1.3*e*, which was 0.7*e* less than their respective formal charges −2*e*. Finally, the net charge of La (+1.7*e*) was 1.3*e* lower than its formal charge of +3*e*. These results agree with those described in other studies [46]. On average, an increase of 0.21*e* was observed in the atomic charge for the three surface oxygen atoms that originated new bonds with the lanthanum ion. The coordinates and the Bader’s charge analysis of the optimized TiO_2_ and La/TiO_2_ surfaces are provided in Table S1 (see Supplementary Materials). According to the literature [34], the results of Bader’s analysis suggest that the La-O bond exhibits a slight ionization due to a possible charge transfer between the lanthanum atom and the oxygen atoms that form the chemical bond [47,48].

**Figure 5:**
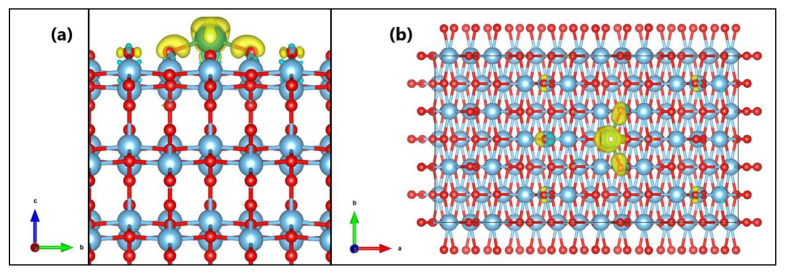
Charge density difference after La-doping on TiO_2._ View from the (**a**) a and (**b**) z axes.

Likewise, to evaluate the charge redistribution on the TiO_2_ surface due to lanthanum doping, a charge difference analysis [47] was used, and the results are shown in Figure 5. The cyan and yellow surfaces shown in the figure correspond to the gain and loss regions, respectively. Therefore, from Figure 5 and the results of Bader’s analysis, it is presumed that the formation of La-O bonds is due to charge transfer between the atoms that form said bonds.

**Figure 6:**
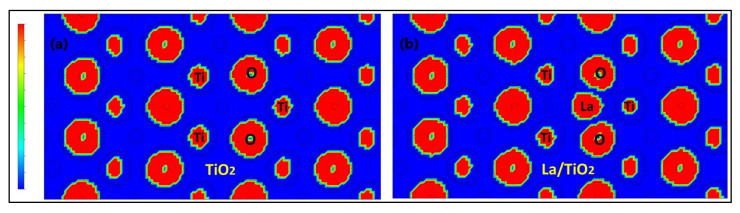
Representation of ELF with contour lines of (**a**) TiO_2_ and (**b**) La/TiO_2_ surfaces, where red means strong electron localization and blue means the opposite.

Additionally, the electron localization function (ELF) was used to better explain the La-O chemical bond based on the region of maximum density (RMD). In ELF analysis, ionic or van der Waals interactions are described by an RMD that is symmetrically distributed around the respective nuclei. Instead, the covalent interaction is described by an RMD that migrates between the nuclei until a completely symmetric geometry is achieved in the ideal covalent case. In Figure 6, the ELF section for the surface (101) of La/TiO_2_ shows the interaction of three surface O atoms with Lanthanum. The figure shows that the RMD of the O nuclei migrates to the junction line with the La nucleus but maintains a slight internuclear separation. In addition, the RMDs do not circumscribe the respective nuclei; therefore, a polar covalent interaction would be generated in the La-O bonds [49,50].

### 2.3. MB Adsorption on Surface (101) of TiO_2_ and La/TiO_2_

**Figure 7:**
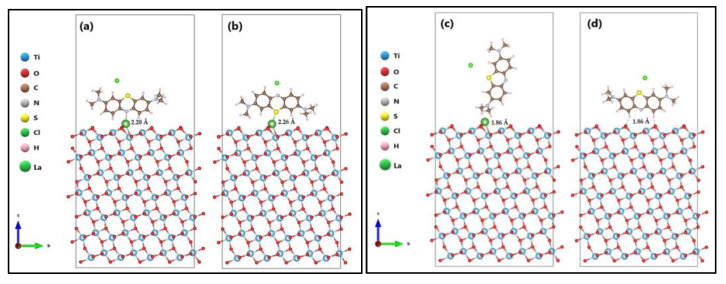
Methylene blue (MB) molecule in (**a**) P_N_, (**b**) P_S_, and (**c**) P_V_ orientations on the La/TiO_2_ surface, and (**d**) P_H_ orientation on the TiO_2_ surface.

As shown in Figure 7, the adsorption of the methylene blue (MB) molecule on the surface (101) of La/TiO_2_ was investigated by placing the MB molecule in the P_N_ and P_S_ orientations, that is, partially parallel to the surface, with the heteroatom (N or S) near the lanthanum. Vertical orientation (P_V_) was also tested with the final methyl groups of the MB molecule oriented towards the lanthanum. Regarding the surface (101) of TiO_2_, preliminary results indicated that the MB molecule oriented parallel to the surface is adsorbed through the methyl groups found at both ends of the molecules (Figure S1, see Supplementary Materials). However, this mode of adsorption allowed the central aromatic ring to bend slightly away from the surface due to electrostatic repulsion between the N and S atoms from the aromatic ring and surface oxygens, which could generate tension in the molecule. Consequently, to clarify the effect of the La^3+^ ion on the MB adsorption capacity of TiO_2_, only the P_H_ orientation shown in Figure 7 was considered to adsorb the MB molecule on the surface (101) of TiO_2_, since the corresponding P_N_ orientation allowed an energetically more favorable adsorption on the La/TiO_2_ surface. 

Figure 7 shows that the MB molecule is adsorbed on the La/TiO_2_ surface by forming a bond between the lanthanum and the respective heteroatom (N or S). The distances from the heteroatom (N or S) of the MB molecule (N_MB_) to the lanthanum on the surface plane of La/TiO_2_ were N_MB_-La_(oxide)_ = 2.20 Å and S_MB_-La_(oxide)_ = 2.26 Å, respectively. Noninteraction was observed when the MB molecule was placed in the P_V_ orientation, although the average distance between the H and the La on the surface plane of La/TiO_2_ was 1.86 Å. On the other hand, the MB adsorption on the TiO_2_ surface was produced by intermolecular forces, probably due to the formation of hydrogen bonds. The average distance from the H atoms of the MB molecule to the surface plane of TiO_2_ was H_MB_-O_(oxide)_ = 1.86 Å. 

To investigate the influence of La^3+^ on molecular adsorption stability, adsorption values ($\Delta E_{ads}$) were calculated. The $\Delta E_{ads}$ of the MB molecule on the surface (101) of both TiO_2_ and La/TiO_2_ oxides was calculated using Equation (2), where $E_{sorb/surf}$ is the energy of the supersystem generated by the MB molecule on the surface of the respective oxide (eV), $E_{surf}$ is the energy of each oxide (eV), and $E_{sorb}$ is the energy of the isolated MB molecule in a vacuum (eV). Likewise, Equation (3) was used to calculate the heat segregation ($\Delta G_{seg}$). Table 2 presents the adsorption energy ($\Delta E_{ads}$) and heat segregation ($\Delta G_{seg}$) of the respective relaxed surfaces.

The adsorption energy value of the MB molecule on the TiO_2_ surface was −95.30 kJ/mol. With the addition of La on the semiconductor surface, the adsorption of the MB molecule through the N-heteroatom of its aromatic ring was more stable (−270.14 kJ/mol). In addition, the heat segregation value for MB adsorption on the La/TiO_2_ surface through both the N and S heteroatoms of the MB ring was calculated to be −58.19 eV and −57.00 eV, respectively. Negative values suggest that the incorporation of La on the semiconductor surface is thermodynamically stable. Therefore, it can be assumed that the presence of La increases the surface binding strength with more stable adsorption energy.

On the other hand, Bader’s charge analysis was also used in order to semi-quantitatively evaluate the charge transfer due to the adsorption process of the MB molecule only in the P_N_ and P_H_ orientations. The results of this analysis for certain atoms at the surface of the semiconductors are listed in Table 3.

Table 3 indicates that there is charge depletion on the H and La atoms and charge accumulation on the O and N atoms; therefore, it is evident that charge transfer occurred between the MB molecule and neighboring atoms in the TiO_2_ and La/TiO_2_ surfaces. For the MB-TiO_2_ system, the charge transfer occurred from the MB molecule, towards the surface, while for the MB-La/TiO_2_ system, the charge transfer occurred in the opposite direction. 

## 3. Discussion

### 3.1. Optimization of La/TiO_2_

The electronic configuration of lanthanum is described as [Xe]4*f*^0^5*d*^1^6*s*^2^. This electronic configuration ensures the properties of lanthanum due to the distribution of electrons with complete levels up to 5, like xenon, but with incomplete level 4*f* and protected by the 6*s* and/or 5*d* orbitals. According to the literature, these *f* orbitals have the capacity to produce complexes with various Lewis bases, increasing the concentration of these species on the surface of various semiconductor oxides and, consequently, the photocatalytic activity [51].

The lanthanum ion used as a dopant in the TiO_2_ semiconductor allows its surface area to be increased because it reduces the growth of the crystalline structure by limiting direct contact between adjacent crystallites [52]. In addition, this ion can increase the thermal stability of TiO_2_, since it interferes with the conversion of the anatase phase to rutile during calcination of the doped semiconductor [53,54,55].

In addition to these advantages, one of the most important characteristics of TiO_2_ doping with La^3+^ is the reduction in the band gap, which optimizes the ability of the semiconductor to absorb wavelengths with less energy, reducing the recombination of vacant/electron pairs and modifying the adsorption capacity of this photocatalyst surface. Another advantage of doping TiO_2_ with La^3+^ is the decrease in photogenerated charge recombination as the photogenerated electrons migrate and are captured by the metal particles, which become active sites for oxygen reduction [56].

Lanthanum in its ionic form (La^3+^) has the ability to generate surface defects and titanium and oxygen vacancies. This possibly occurs because La^3+^ (1.15 Å) has a larger atomic radius than Ti^4+^ (0.68 Å) [57,58]. Consequently, the La^3+^ ion cannot replace the Ti^4+^ ions in the semiconductor lattice, so it only adsorbs on the surface, forming Ti–O–Ln^3+^ bonds with ionic/covalent characteristics [44], leading to an imbalance in the surface charges of the crystalline structure of TiO_2_.

Since the titanium atom has a higher Pauling electronegativity value (1.54) than lanthanum atoms (1.10), there is a transfer of electrons from lanthanum to titanium, which changes from Ti^4+^ to Ti^3+^. The excess negative charge disrupts the electronegativity, since it is necessary to remove an O^2−^ ion for every two Ti^3+^ ions produced, which promotes the formation of oxygen and titanium vacancies, causing a surface defect. Moreover, the presence of La^3+^, dispersed as interstitial impurities in the TiO_2_ lattice, generates surface defects. Due to the presence of these vacancies and defects, the electrons produced on the photocatalyst’s surface are captured, and therefore, the recombination of photogenerated charge carriers is diminished. However, an excess of La^3+^ on the semiconductor surface reduces its photocatalytic capacity since it generates a high density of defects and vacancies, which act as recombination centers instead of electron collectors [51].

In a previous experimental study, we demonstrated the specific effect of lanthanum on the inhibition of TiO_2_ crystallite growth, as well as the stabilization of the anatase phase relative to the rutile phase, under the applied synthesis conditions [54]. In this theoretical study, we use a model with a La ion placed on the upper layer of TiO_2_ to explain the binding mode of this chemical element on the semiconductor surface. In Figure 1, the optimized surfaces (101) of TiO_2_ and La/TiO_2_ are shown comparatively. In this figure, the formation of La-O-Ti bonds on the TiO_2_ surface can be observed, which supports the experimental results reported in the literature [56,59,60,61].

The formation of La-O-Ti bonds, in addition to stabilizing small crystalline particles [52], can produce a change in the band structure and density of states of the surface electrons [62]. Therefore, to also verify this hypothesis, the DFT calculation was performed using the optimized La/TiO_2_ model.

### 3.2. Electronic Structure of La/TiO_2_

The indirect bandgap value of La/TiO_2_ shown in Figure 2 was determined by implementing a Hubbard approximation term in order to accurately explain the electronic structure of this semiconductor [59]. In this theoretical study, the value of the indirect bandgap for the La/TiO_2_ structure was lower than the value obtained for the TiO_2_ structure, and also lower than the value we reported in a previous study [7]. Therefore, according to the literature, La/TiO_2_ could be more photoactive than TiO_2_ due to the smaller separation between the occupied and unoccupied bands [63,64]. In fact, in a previous experimental study, we demonstrated that the narrowing of the bandgap due to the incorporation of the La ion is beneficial to broaden the edge of the absorption band of a photocatalyst in the visible light region [54,55].

Table 4 shows the bandgap energy difference between TiO_2_ and La/TiO_2_ calculated in this study compared to other values reported in the literature.

On the other hand, the electronic states of La/TiO_2_, Figure 3 and Figure 4, show that the hybridization of Ti-O bonds occurs mainly in their 2*p* and 3*d* orbitals, while the hybridization of the La-O bond occurs mainly in their 2*p* and 5*d* orbitals. The existence of these hybrid energy levels can help as recombination centers for photogenerated electrons and holes. Thus, the competitive effect of the photogenerated (*e*^−^/*h*^+^) pair recombination and the narrow bandgap that promotes absorption in the visible region co-affects the photocatalytic efficiency of La/TiO_2_. The charge transfer of the Ti-O and La-O bonds shows that the empty 3*d* orbital in Ti^4+^ (3d^0^) forms a covalent bond with the O2*p* orbital on the respective O atoms, while the nearly empty 5*d* orbital in La^3+^ (5*d*^1^) forms a covalent bond slightly ionized with the O2*p* orbital on the respective O atoms.

In this study, it was verified that the electronic structure of the TiO_2_ surface undergoes a change with the incorporation of the La^3+^ ion. Therefore, ELF analysis was used for a better description of the La-O chemical bond on the La/TiO_2_ surface. According to the literature, the charge-depletion zones around the La atom and the charge-accumulation zones around the three O atoms shown in Figure 6 could suggest ionic bonds between the La and O atoms [44]. However, in the same figure, the symmetrical contours around the La and O atoms demonstrate the separation of these still polarized atoms, suggesting the formation of a polar covalent bond [49].

### 3.3. MB Adsorption on Surface (101) of TiO_2_ and La/TiO_2_

The large diameter of the La ion is useful for enhancing the specific surface area (SSA) of TiO_2_, while the strong electron-withdrawing effect that this ion possesses [30] may contribute to the formation of Lewis acidic sites that provide stability to the semiconductor in an aqueous reaction medium [67,68]. The formation of these acidic sites on the TiO_2_ surface allows the effective adsorption of organic contaminants [57,69], such as methylene blue (MB) dye [55].

Numerous experimental studies of MB dye removal from aqueous solutions have confirmed that this dye can be easily adsorbed on the surface of a semiconductor oxide by electrostatic attraction between the positive regions of the MB molecule and the oxygen atoms on the semiconductor surface [8]. In fact, a study by Khnifira et al. [70] shows that the adsorption of MB in its neutral and protonated forms on the surface (110) of anatase occurs via hydrogen bond formation and is more stable when the dye molecule is oriented parallel to the surface of the semiconductor. On the other hand, in a previous study, we demonstrated that for the surface (101) of anatase, adsorption occurs in a bidentate chelating mode through hydrogen bonds formation and is more stable when the dye molecule is oriented perpendicular to the semiconductor surface [7]. Therefore, it is evident that in the adsorption process, the orientation of the adsorbate molecule with respect to the adsorbent surface will depend on the chemical nature of said surface. In this study, in which the surface (101) of anatase was also considered, a better adsorption of MB dye was evidenced when the molecule was placed with a partially parallel orientation and with the N heteroatom close to the semiconductor surface. The adsorption energy value determined in this study for the La/TiO_2_ surface with the MB molecule placed in P_N_ orientation (*E_ads_* = −270.14 kJ/mol) proved to be more negative compared to the energy value determined for the P_S_ orientation (*E_ads_* = −112.75 kJ/mol). According to the literature, lateral adsorbate–adsorbate interactions, including electrostatic repulsion, could have an important effect on the molecular adsorption force [71]. Consequently, the results of this study suggest that the adsorption process of the MB molecule on the surface (101) of the semiconductor oxide would be determined by the orientation of the molecule with respect to the surface, which would result from the balance between the lateral MB-MB interactions.

In addition, the results of this theoretical study show that the molecular adsorption process of MB on the (101) surface of La/TiO_2_ is energetically more favorable than on the (101) surface of TiO_2_ (*E_ads_* = −95.30 kJ/mol). Therefore, it is suggested that lanthanum doping could improve the adsorption capacity of TiO_2_.

#### Proposed Photocatalytic Mechanism

In this study, La/TiO_2_ allowed a more effective adsorption of the methylene blue molecule compared to TiO_2_. This is probably because the bonding of the La ion on the TiO_2_ surface could provide more active adsorption centers for the dye molecule due to the formation of Lewis acid sites. On the other hand, from the photocatalytic photodegradation results obtained in a previous experimental study, and based on the electronic structure results obtained in this theoretical study, it is suggested that the bonding of the La ion on the TiO_2_ surface also influences the photocatalytic activity of this semiconductor. This is probably because the presence of the La ion on the surface of TiO_2_ not only reduces the bandgap energy of this semiconductor but could also enhance the transfer of photoinduced electrons from the bulk to the catalyst surface and thus prevent the recombination of (*e^−^/h^+^*) pairs under the effect of radiation [38].

Since it has been suggested that the La ion influences the photocatalytic activity of TiO_2_ by altering the recombination rate of the (*e*^−^/*h*^+^) pair, and the photocatalytic process involving the La/TiO_2_ catalyst could occur with the formation of an electron/hole pair (*e*^−^/*h*^+^) because the electrons (*e*^−^) of the photocatalyst are photoexcited and transferred from the valence band (VB) to the conduction band (CB), leaving a hole (*h*^+^) in the VB (reaction R1). The photogenerated pairs (*e*^−^/*h*^+^) can immediately recombine (reaction R2); however, some of these pairs can migrate to the semiconductor surface to react separately with species adsorbed on the catalyst surface, such as H_2_O, OH^−^, O_2_, and other molecules (R), including the MB dye. The holes (*h*^+^) generated in the VB of the catalyst can oxidize adsorbed water molecules or hydroxyl ions to generate strongly reactive hydroxyl radicals (reactions R3 and R4). Furthermore, these holes could diffuse to the TiO_2_ surface to produce more reactive radicals that oxidize adsorbed molecules on the surface. On the other hand, the empty 4*f* and 5*d* orbitals of the La ion can host photoexcited electrons in the CB of TiO_2_ (reaction R5); therefore, the La^3+^ ion can capture an electron and be reduced to the La^2+^ ion. The instability of this ion would allow the captured electrons to be transferred to the oxygenated molecules adsorbed on the TiO_2_ surface, generating superoxide radical anions (O_2_^−^) and hydroxyl radicals (OH) through a sequence of reactions (reactions R6–R9). These radicals have a great capacity to oxidize organic molecules. The O_2_^−^ can oxidize organic compounds with strong electron-donating groups, while the OH radical can remove H atoms or attack unsaturated C-C bonds [72]. Consequently, it is very likely that a molecule containing unsaturated C-C bonds, such as MB, will be photo-oxidized or attacked by the OH radicals formed in the photocatalytic pathway (reaction R10). On the other hand, immediate oxidation of organic compounds is also possible if the molecules react directly with the photogenerated holes (reaction R11) [52]. The following reactions suggest the likely route for MB dye photodegradation on the La/TiO_2_ surface [60]

(R1) $$\left(TiO_{2}\right)\overset{hv}{\to }\;TiO_{2}+e_{CB}^{-}+h_{VB}^{+}$$

(R2) $$e_{CB}^{-}+h_{VB}^{+}\to heat$$

(R3) $$H_{2}O_{ads}+h_{VB}^{+}\;⇌\;{\left(H^{+}+OH^{-}\right)}_{ads}+h_{VB}^{+}\to OH_{ads}^{•}$$

(R4) $$OH_{ads}^{-}+h_{VB}^{+}\to \;OH_{ads}^{•}$$

(R5) $$La^{3+}+e_{CB}^{-}\;\to \;La^{2+}$$

(R6) $$La^{2+}+{\left(O_{2}\right)}_{ads}\;\to \;La^{3+}+O_{2}^{•-}$$

(R7) $$O_{2}^{•-}+H^{+}\to \;HO_{2}^{•}$$

(R8) $$2HO_{2}^{•}\to \;H_{2}O_{2}+O_{2}$$

(R9) $$H_{2}O_{2}+e_{BC}^{-}\to \;OH^{•}+OH^{-}$$

(R10) $$R+OH_{ads}^{•}\to \;R_{ads}^{\prime •}+H_{2}O\to degradation\;products$$

(R11) $$R_{ads}+h_{VB}^{+}\to \;R_{ads}^{•+}\to degradation\;products$$

## 4. Materials and Methods

Calculations were performed at the density functional theory (DFT) level using the Vienna Ab Initio Simulation Package (VASP) version 6.0 (VASP Software GmbH, Vienna, Austria) [73,74,75]. The construction and visualization of the molecular models were carried out in the molecular modeling program BioVia Materials Studio, version 5.5 (BioVia, San Diego, CA, USA). All periodic DFT calculations were carried out using the projector augmented wave (PAW) approach [76]. We adopted the GGA-PBE exchange-correlation functional to describe electronic exchange–correlation interactions [77]. A plane wave cutoff energy of 500 eV was used in all calculations. The Brillouin zone was sampled using Γ-centered Monkhorst-Pack *k*-point meshes (1 × 3 × 1) [78]. A convergence energy criterion of 10^−5^ eV was imposed on the self-consistent cycles to solve the Kohn–Sham equations [79]. Geometry optimizations were carried out until the maximum residual forces were less than 0.005 eV/Å. To improve the convergence of the total energy, the Gaussian smearing method was used with σ = 0.10 eV. All calculations were non-spin-polarized. The functional GGA + U was used in the calculation of the electronic properties [80]. Hubbard U values were established at 2.5 eV and 6.0 eV for Ti and La atoms, respectively [33,35]. The adsorption of the MB molecule on the anatase surface was modeled using the following previously optimized parameters: tetragonal TiO_2_ with a cell = 3.82 Å × 3.82 Å × 9.70 Å <90° × 90° × 90°> [81]. For the lanthanum anchoring and MB adsorption studies, the bulk of TiO_2_ was cleaved at the stable surface (101) [82,83,84]. The TiO_2_ and La/TiO_2_ surfaces (101) consisted of seven semiconductor atomic layers with a p(3 × 3) supercell, containing 168 Ti atoms, 336 O atoms and 1 La atom on the doped surface. The cell size was selected by looking for the best compromise between computational accuracy, computational cost, and minimization of lateral MB-MB interactions. From the periodic boundary conditions (PBC) incorporated in the DFT calculation, the average distance between MB molecules and their nearest neighbors was estimated to be 5.48 Å and 7.82 Å for the horizontal and vertical orientations, respectively. 

The surface energies ($\gamma_{s}$) of the La/TiO_2_ structure with a vacuum distance of 20 Å were calculated using the following equation [44,85]:

(1) $$\gamma_{s}=\;\frac{\left(E_{slab}-n\times E_{bulk}\right)}{2A}$$

where $E_{slab}$ is the total energy of the slab material (eV), $E_{bulk}$ is the total energy of the bulk material (eV), n is the number of atoms involved in the slab, and A is the surface area (Å^2^).

On the other hand, the adsorption energy ($\Delta E_{ads}$) of the MB molecule on the surface (101) of both TiO_2_ and La/TiO_2_ oxides was calculated using the following equation [33,86]:

(2) $$\Delta E_{ads}=E_{sorb/surf}-E_{surf}-E_{sorb}$$

where $E_{sorb/surf}$ is the energy of the supersystem produced by the adsorbed molecule on the surface (eV), $E_{surf}$ is the energy of the surface (eV), and $E_{sorb}$ is the energy of the isolated molecule in a vacuum (eV).

Furthermore, to investigate the influence of La on the molecular adsorption stability, values of heat of segregation ($\Delta G_{seg}$) relaxation were calculated. The heat segregation ($\Delta G_{seg}$) was calculated using the following equation [33]:

(3) $$\Delta G_{seg}\;=\;\frac{1}{n}(E_{MB/oxide:nLa}-E_{MB/oxide}-n\mu_{Het}+n\mu_{La})$$

where $E_{MB/oxide:nLa}$ and $E_{MB/oxide}$ are the total energies of the surfaces with and without La, n is the number of the La atoms on the surface, μ is the chemical potential of the heteroatom (N or S) of the MB ring. In general, a more negative value of $\Delta G_{seg}$ evidences that the surface is thermodynamically more stable.

## 5. Conclusions

Anatase (TiO_2_) is a semiconductor with a wide bandgap of 3.2 eV, so the biggest problem with the use of this oxide for photocatalytic applications lies in the fact that it is only active under ultraviolet light radiation. The incorporation of Lanthanum (La) on the TiO_2_ surface could solve this problem, since it allows the formation of hybrid levels in the VB and CB, changes the states of the surface electrons, reduces the potential energy of molecular adsorption, and reduces the energy barriers (bandgap) for photocatalytic processes under solar radiation. The incorporation of La on the surface of anatase also distorts the surface lattice of this photocatalyst, which inhibits crystallite growth to obtain nanoparticles with a higher specific surface area.

Experimental evidence indicates that the La/TiO_2_ photocatalyst exhibits better MB removal capacity from aqueous solutions than the TiO_2_ photocatalyst. Therefore, in this theoretical study, DFT calculations were performed to clarify the influence of La on the electronic properties of TiO_2_, as well as its ability to remove MB dye. The incorporation of La on the TiO_2_ surface proved to be energetically stable due to the formation of polar covalent La-O bonds, which had both ionic and covalent characteristics. Furthermore, in this study the lower bandgap energy of La/TiO_2_ was verified compared to the bandgap energy of TiO_2_, suggesting that semiconductor La/TiO_2_ could be more efficient than TiO_2_ for MB dye photodegradation. On the other hand, the results of this study also demonstrate that the incorporation of the La^3+^ ion in the TiO_2_ semiconductor improves its MB adsorption capacity. Therefore, according to the literature and the evidence of this study, the La/TiO_2_ photocatalyst could be more efficient than TiO_2_ for MB dye removal. The elimination process would begin with the adsorption of the MB molecule on the La/TiO_2_ surface, which occurs at room temperature and in the absence of radiation. Then, the electrons in the VB of La/TiO_2_ can be photoexcited to hybrid levels through the band transition [33]. Likewise, the electrons in the CB can be photoexcited to the photocatalyst surface through the intra-band transition to finally oxidize the methylene blue molecule.

In conclusion, the results of this theoretical study demonstrate that the semiconductor La/TiO_2_ can efficiently combine adsorption and photocatalysis processes for the effective removal of methylene blue dye. This study lays the theoretical foundations for the practical technological applications that this semiconductor material could have in the field of environmental remediation and effluent treatment and motivates further studies on the adsorption of other dyes in this matrix.

## Figures and Tables

**Table 1:** Significant bond lengths and angles for La/TiO_2_ surface.

Atoms	Bond Length (Å)	Atoms	Angle (°)
La-O62	2.19	La-O62-Ti87	92.95
		La-O62-Ti86	114.27
La-O66	2.19	La-O66-Ti143	92.96
		La-O66-Ti142	114.29
La-O178	2.43	La-O178-Ti143	90.30
		La-O178-Ti57	91.13
		La-O178-Ti87	91.97

**Table 2:** Adsorption energy and heat segregation for different surfaces.

Position	Bond	∆*E_ads_* (kJ/mol)	∆*G_seg_* (kJ/mol)
La/TiO_2_ (P_N_)	La-N	−270.14	−58.19
La/TiO_2_ (P_S_)	La-S	−112.75	−57.00
La/TiO_2_ (P_V_)	-	+78.35	-
TiO_2_ (P_H_)	O-H	−95.30	-

**Table 3:** Bader’s charge analysis for the selected atoms at the surfaces.

Absorption System	Atom	Total Electron (−*e*) before Adsorption	Total Electron (−*e*) after Adsorption	Transfer Charge (−*e*)
MB absorbed on La/TiO_2_ (P_N_)	N1(523)	7.2142	7.6493	−0.4351
	La1(505)	9.2964	9.1019	+0.1945
MB absorbed on TiO_2_ (P_H_)	H6(531)	0.8447	0.8098	+0.035
	H2(527)	0.7363	0.7241	+0.012
	O66(234)	7.1144	7.1951	−0.0807
	O59(227)	7.1574	7.2155	−0.0581

**Table 4:** Bandgap energy differences (eV) between TiO_2_ and La/TiO_2_.

Adsorbent	Bandgap Energy (eV)	Energy Difference (eV)	Method	Reference
TiO_2_	3.10	0.18	Experimental	[52]
La/TiO_2_	2.92			
TiO_2_ (rutile)	1.94	0.15	CASTEP (GGA/PBE)	[64]
La/TiO_2_ (rutile)	1.79			
TiO_2_ (anatase)	3.20	0.70	Quantum ESPRESSO (LDA + U)	[65]
La/TiO_2_ (anatase)	2.50			
TiO_2_ (anatase)	2.72	0.38	VASP (GGA/PBE + U)	[66]
La/TiO_2_ (anatase)	2.34			
TiO_2_ (anatase)	3.21	0.23	VASP (GGA/PBE + U)	[7]
La/TiO_2_ (anatase)	2.98			This study

## Data Availability

Data are contained within the article and Supplementary Materials.

## Supplementary Materials

The following supporting information can be downloaded at: https://www.mdpi.com/article/10.3390/molecules27196370/s1, Figure S1: Aromatic ring of MB bent slightly on the surface (101) of TiO_2_. Table S1: Bader’s charge analysis of the optimized MB molecule, free and adsorbed, on TiO_2_ and La/TiO_2_ surfaces.
