# Supplementary material for: La3+’s Effect on the Surface (101) of Anatase for Methylene Blue Dye Removal, a DFT Study
Source: Molecules. 2022 Sep 27;27(19):6370. doi: 10.3390/molecules27196370 (PMC9571724; doi:10.3390/molecules27196370)
Supplement: Supplementary file 1 [file molecules-27-06370-s001.zip › molecules-1925513-supplementary.pdf]

molecules

MDPI

Supplementary Material

La^{3+} Effect on the Surface (101) of Anatase for Methylene Blue Dye Removal, a DFT Study

Ximena Jaramillo-Fierro *, Sneyder Gaona and Eduardo Valarezo

Departamento de Química, Facultad de Ciencias Exactas y Naturales, Universidad Técnica Particular de Loja, San Cayetano Alto, Loja 1101608, Ecuador
* Correspondence: xvjaramillo@utpl.edu.ec; Tel.: +593-7-3701444

Abstract: Density functional theory (DFT) is a widely used method for studying matter at the quantum level. In this study, the surface (101) of TiO_2 (anatase phase) was considered to develop DFT calculations and explain the effect of lanthanum ion (La^{3+}) on the electronic properties, adsorption capacity, and photocatalytic activity of this semiconductor. Due to the presence of the La^{3+} ion, the bandgap energy value of La/TiO_2 (2.98 eV) was lower than that obtained for TiO_2 (3.21 eV). TDOS analysis demonstrated the presence of hybrid levels in La/TiO_2 composed mainly of O2*p* and La5*d* orbitals. The chemical nature of the La-O bond was estimated from PDOS analysis, Bader charge analysis, and ELF function, resulting in a polar covalent type, due to the combination of covalent and ionic bonds. In general, the adsorption of the methylene blue (MB) molecule on the surface (101) of La/TiO_2 was energetically more favorable than on the surface (101) of TiO_2. The thermodynamic stability of doping TiO_2 with lanthanum was deduced from the negative heat-segmentation values obtained. The evidence from this theoretical study supports the experimental results reported in the literature and suggests that the semiconductor La/TiO_2 is a potential catalyst for applications that require sunlight.

Keywords: DFT calculations; anatase; lanthanum; adsorption; methylene blue

Citation: Jaramillo-Fierro, X.; Gaona, S.; Valarezo, E. La3+'s Effect on the Surface (101) of Anatase for Methylene Blue Dye Removal, a DFT Study. *Molecules* **2022**, *27*, 6370. https://doi.org/10.3390/molecules27196370

Academic Editors: Pierfrancesco Cerruti and Arash Moeini

Received: 3 September 2022
Accepted: 23 September 2022
Published: 27 September 2022

Publisher's Note: MDPI stays neutral with regard to jurisdictional claims in published maps and institutional affiliations.

Copyright: © 2022 by the authors. Licensee MDPI, Basel, Switzerland. This article is an open access article distributed under the terms and conditions of the Creative Commons Attribution (CC BY) license (https://creativecommons.org/licenses/by/4.0/).

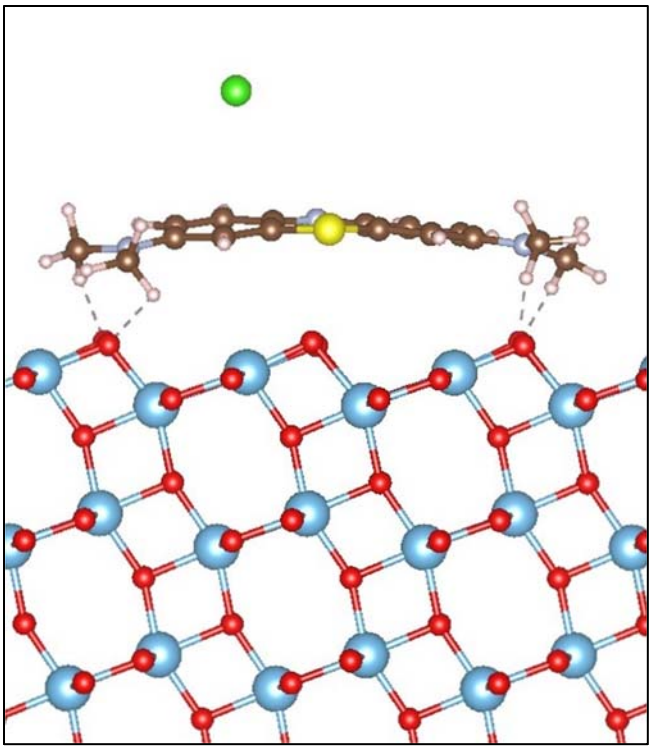

Figure S1. Aromatic ring of MB bent slightly on the surface (101) of TiO_2.

Table S1. Bader`s charge analysis of the optimized MB molecule free and adsorbed on TiO_2 and La/TiO_2 surfaces.

No.	Atom	TiO_2 surface			La/TiO_2 surface		
		MB Charge (-*e*)	MB-TiO_2 Charge (-*e*)	Difference Charge (-*e*)	MB Charge (-*e*)	MB-La/TiO_2 Charge (-*e*)	Difference Charge (-*e*)
**1**	C	−0.072	−0.016	0.056	−0.979	0.013	0.992
2	C	1.195	1.044	−0.151	1.275	1.094	−0.181
3	C	1.377	−0.034	−1.411	0.771	0.115	−0.656
4	C	−0.214	−0.097	0.117	−0.026	−0.149	−0.123
5	C	−1.515	−0.062	1.454	−1.446	−0.321	1.126
6	C	1.164	1.336	0.172	0.822	1.107	0.285
7	C	−0.301	−0.294	0.007	−0.251	−0.201	0.050
8	C	−0.022	0.094	0.116	−0.710	0.208	0.918
9	C	1.186	1.088	−0.098	0.860	0.912	0.053
10	C	−0.063	−0.202	−0.140	−0.931	−0.055	0.876
11	C	−0.256	0.162	0.418	0.026	0.182	0.156
12	C	1.128	1.352	0.225	0.092	1.085	0.993
13	C	0.175	0.689	0.515	0.053	0.738	0.685
14	C	0.209	0.716	0.507	0.005	0.658	0.653
15	C	0.271	0.616	0.346	0.240	0.740	0.501
16	C	0.382	0.778	0.396	0.260	0.789	0.529
17	S	0.575	0.267	−0.308	0.618	0.306	−0.312
18	N	−2.373	−2.674	−0.301	−2.214	−2.649	−0.435
19	N	−2.301	−2.417	−0.116	−2.025	−2.452	−0.427
20	N	−2.277	−2.431	−0.154	−2.406	−2.530	−0.124
21	CL	−0.942	−0.374	0.567	−0.789	−0.629	0.160

22	H	0.215	0.090	−0.124	1.000	0.100	−0.900
23	H	0.264	0.276	0.012	0.122	−0.031	−0.153
24	H	0.283	0.041	−0.243	1.000	0.185	−0.815
25	H	0.307	0.077	−0.231	1.000	0.027	−0.974
26	H	0.158	0.058	−0.100	0.999	0.051	−0.948
27	H	0.155	0.190	0.035	0.994	−0.096	−1.090
28	H	0.173	0.024	−0.149	0.195	0.024	−0.171
29	H	0.161	0.047	−0.114	0.134	−0.004	−0.138
30	H	0.213	0.032	−0.181	0.166	0.058	−0.108
31	H	0.107	0.009	−0.098	0.131	0.056	−0.076
32	H	0.123	0.037	−0.086	0.152	0.047	−0.105
33	H	0.071	0.060	−0.011	0.158	0.033	−0.125
34	H	0.024	0.065	0.042	0.063	0.015	−0.049
35	H	0.101	0.044	−0.056	0.090	0.048	−0.042
36	H	0.048	0.080	0.032	0.098	0.033	−0.065
37	H	0.098	0.056	−0.042	0.176	0.030	−0.146
38	H	0.104	0.040	−0.064	0.122	−0.001	−0.123
39	H	0.069	−0.030	−0.099	0.155	0.029	−0.126
